# Genetic and Phenotypic Characterization of *Bacillus velezensis* Strain BV379 for Human Probiotic Applications

**DOI:** 10.3390/microorganisms12030436

**Published:** 2024-02-21

**Authors:** Laura M. Brutscher, Sebhat Gebrechristos, Sean M. Garvey, Jessica L. Spears

**Affiliations:** 1 BIO-CAT Microbials, LLC, Shakopee, MN 55379, USA; 2 BIO-CAT, Inc., Troy, VA 22974, USA

**Keywords:** probiotic, *Bacillus velezensis*, BV379, safety, toxicology, endospore

## Abstract

Bacterial spore-forming *Bacillaceae* species, including *Bacillus subtilis* and *Heyndrickxia coagulans*, are increasingly utilized for probiotic dietary supplementation. *Bacillus velezensis* is a *Bacillus* species that is frequently used as a direct-fed microbial in animal feed but less so as a probiotic for humans. The objective of this study was to characterize the suitability of the *Bacillus velezensis* strain BV379 for probiotic applications by (1) in silico screening for both adverse genetic elements and putatively beneficial traits, (2) in vitro evaluation of interactions with human intestinal epithelial cells, and (3) in vitro characterization of BV379 spore viability at various temperatures, pH, and in the presence of bile salt. In silico screening of the BV379 genome revealed few genes encoding *Bacillaceae*-associated toxins, virulence factors, and enzymes involved in the production of toxins. While BV379 encodes five antimicrobial resistance genes, minimum inhibitory concentration assays determined that BV379 is susceptible to all eight clinically relevant antibiotics tested. Preliminary cell culture experiments showed that BV379 lysates did not adversely impact human intestinal epithelial cell viability and monolayer permeability. It was also determined that BV379 spores can easily tolerate the harsh pH, bile salt, and microaerobic conditions typical of the GI tract. Altogether, the results presented herein support the safety and potential of *Bacillus velezensis* strain BV379 for use as an oral probiotic.

## 1. Introduction

Members of the bacterial taxonomic family *Bacillaceae* are some of the most widely distributed microorganisms in nature, participating in a variety of biochemical processes across ecologically diverse environments [1,2,3,4]. For over half a century, biotechnology industries including pharmaceuticals, nutraceuticals, and agriculture have leveraged *Bacillaceae* strains for their aptitude for protein secretion, metabolic versatility, and robust growth in relatively short fermentation periods [5,6,7,8]. In addition, *Bacillaceae* strains are able to form spores that exhibit resistance to a variety of environmental stressors [7,9].

Spore-forming *Bacillaceae* species have also been increasingly used for both animal and human probiotic applications [10,11,12]. Probiotics are defined as live microorganisms that confer a health benefit to the host when administered in adequate amounts [13]. Studies have shown the roles of probiotics in gastrointestinal (GI) tract maintenance, gut immunity, dysbiosis, and extraintestinal biology, such as mood and cognition [14,15]. While established probiotic strains from the bacterial *Lactobacillaceae* family and *Bifidobacterium* genus and fungal strains from the *Saccharomyces* genus may require protective microencapsulation to survive transit through the GI tract [16,17,18], spore-forming *Bacillaceae* species are generally more resistant to the harsh microenvironments of the GI tract, including acidic gastric pH, intestinal hypoxia, bile salts, and dense colonization by resident microbes [19]. Commercial probiotic strains of *Bacillus subtilis* and *Heyndrickxia coagulans* (formerly *Bacillus coagulans*) have been clinically shown to support overall GI tract health and nutrient absorption in healthy adults [20,21,22,23] and reduce GI symptoms in individuals with mild digestive discomfort [20,21,24,25,26,27,28,29,30], dyspepsia [31,32], or inflammatory bowel syndrome [33,34,35,36,37]. Additionally, *Alkalihalobacillus clausii* (formerly *Bacillus clausii*) strains have been demonstrated to modulate immunity in several clinical trials [38,39,40]. 

*Bacillus velezensis* is a recently recognized *Bacillaceae* species and emerging candidate probiotic. First isolated from the Vélez River in Málaga, Spain, and characterized in 2005 [41], *B. velezensis* is widely distributed in nature and well known for antifungal, antibacterial, and plant growth-promoting and biocontrol properties in the soil rhizosphere of crops [42,43]. *B. velezensis* strains have also been isolated from traditionally fermented foods, such as douchi [44,45], soy sauce [46,47], fish sauce [48], fermented soybean (*meju* and *doenjang*) [46,49], sea squirt (munggae) jeotgal [50,51], and kimchi [52,53], demonstrating their natural occurrence in foods. Fermentation-derived *B*. *velezensis* strains are being developed for use as fermentation starter strains in soy bean fermentation, as well as direct formulation into functional foods products (e.g., chocolate) [49,54,55,56].

Importantly, several *B. velezensis* strains (e.g., strains LOH112, ADS024, Marseille-Q1230, MV4, and MV11) have been isolated from human stool and cervicovaginal samples, suggesting that *B. velezensis* naturally occurs in the human gut and vaginal microbiotas [57,58,59,60]. *B. velezensis* strains secrete a wide array of bioactive molecules, including antimicrobial metabolites such as macrolactin, antimicrobial lipopeptides like fengycin, and digestive enzymes [61]. For this reason, *B. velezensis* strains have also been extensively and safely utilized as probiotics in animal feed and aquaculture additives [62,63,64,65,66,67,68]. Accordingly, *B. velezensis* has been listed by the European Food Safety Authority (EFSA) in the approved QPS (Qualified Presumption of Safety) list [69,70]. In humans, two long established probiotic strains, *B*. *polyfermenticus* SCD (Bispan) and *B*. *subtilis* C-3102 (also known as DSM 15544 or Calsporin^®^), which were recently reclassified as *B*. *velezensis* strains [71,72], were shown to be safe for oral consumption up to 4.8 × 10^10^ CFU/day across four human clinical trials [73,74,75,76]. Thus, the genus *B*. *velezensis* remains a promising pool from which to develop novel and differentiated probiotics. 

The aim of this study was to evaluate a unique *B. velezensis* strain, *B. velezensis* BV379 (BV379), for use in human probiotic applications. To characterize the preclinical safety of BV379, the genome was screened in silico for the presence of genetic elements encoding *Bacillaceae*-associated toxins, secondary metabolites, virulence factors, antibiotic resistance, and mobile gene elements that could facilitate horizontal transfer of antibiotic resistance genes. In vitro minimum inhibitory concentration (MIC) assays were utilized to further understand antibiotic resistance. The effects of BV379 lysates on human intestinal epithelial cell viability and cell membrane permeability were also tested. To characterize the probiotic potential of BV379, spores were tested under various pH, temperature, bile salt, and oxygen conditions to assess the strain’s suitability for use in manufacturing and survivability through the GI tract. 

## 2. Materials and Methods

### 2.1. Strain Isolation and Spore Preparation

*B. velezensis* strain BV379 (ATCC Accession No. PTA-127359, hereinafter referred to as BV379) is a Gram-positive, spore-forming facultative bacterium that was isolated at BIO-CAT Microbials, LLC (Shakopee, MN, USA), from soil collected from Douglas County, WI, USA, on 7 June 2015. Following previously established methods to isolate spore-forming bacteria [77], BV379 was isolated by suspending the soil sample in Butterfield’s buffer and heating the solution to 80 °C for 7 min to enrich spore-forming bacteria. The soil solution was then serially diluted and plated onto trypticase soy agar (TSA) and incubated overnight at 35 °C. BV379 is a product of one of the colonies.

Two batches of spore powder were prepared by culturing BV379 in a yeast-based medium for 42–46 h at 37 °C. Per proprietary manufacturing protocols, the culture was then adjusted to a pH < 5, pelleted, spray-dried, and diluted with maltodextrin to a concentration of ~1 × 10^11^ CFU/mL. 

### 2.2. Genome Sequencing and Annotation

To obtain high molecular weight genomic DNA, BV379 was first grown in tryptic soy broth (TSB) overnight. The Qiagen Genomic Tip 100/G kit (Hilden, Germany) was used to extract DNA from the culture according to manufacturer’s instructions. DNA was further purified using Genomic DNA Clean and Concentrator columns (Zymo Research, Irvine, CA, USA). The DeNovix^®^ dsDNA Broad Range fluorometric assay (DeNovix, Wilmington, DE, USA) was used to determine the DNA quality and quantity. 

The sample was barcoded and sequenced using Native Barcoding kits (EXP-NBD104 and EXP-NBD114) and SQK-LSK109 chemistry from Oxford Nanopore Technologies (Oxford, UK). CleanNGS SPRI (solid-phase reversible immobilization) magnetic beads were used to clean up pooled libraries prior to sequencing (Clean NA, Waddinxveen, the Netherlands). The pooled library was then sequenced on MinION FlowCells (Catalog No. FLO-MIN106D, Oxford Nanopore Technologies, Oxford, UK) for 48–72 h. The BV379 genome was fully assembled with Flye using the default parameters [78]. The assembled genome resulted in a single, circular contig 4,057,103 bp in length with a guanine and cytosine (GC) content of 46.5%. No plasmids were detected. The BV379 genome was uploaded to the Bacterial and Viral Bioinformatics Resource Center (BV-BRC) server and annotated utilizing the RAST tool kit (RASTtk) [79,80]. 

### 2.3. Taxonomic Classification

Local BLASTn from the BLAST+ software suite (version 2.9.0-2) [81,82] was used to identify nucleotide sequences in the BV379 genome and 22 other *B. velezensis* genomes, seven genomes from strains classified as *B*. *amyloliquefaciens*, and three *B*. *subtilis* genomes that aligned with six genes from the genome of *B. velezensis* strain FZB42 (formerly a *B. amyloliquefaciens*-type strain): *rpoB* (GeneID: 46872094), *purH* (GeneID: 46871554), *gyrA* (GeneID: 46872224), *groEL* (GeneID: 46871600), *polC* (GeneID: 46870591), and *16S rRNA* (GeneID: NR_075005.2). These sequences are common “housekeeping” genes used for phylogenetic differentiation of *Bacillus* species. For each genome, the nucleotide sequences matching the housekeeping genes were combined into one sequence (~15,100 nt). Comparator strains with complete genome assemblies were chosen from the National Center for Biotechnology Information (NCBI) database. The partially assembled genome of the probiotic strain *B*. *velezensis* GF423 was also included as a comparator genome [72]. 

The online program MAFFT (Multiple Alignment using Fast Fourier Transform) was used to perform a multiple sequence alignment for the concatenated sequences from each *Bacillus* strain [83]. The output MAFFT alignment file was uploaded into MEGA X, and the maximum likelihood method and Tamura–Nei model were used for phylogenetic tree construction [84,85]. After bootstrapping the data 50 times, the tree with the highest log likelihood (−28,334.99) was selected. There were a total of 15,095 positions in the final dataset. 

The sequence identities between the whole genome of BV379 and the other genomes were further determined by performing pairwise average nucleotide identity (ANI) analyses via the web server jSpeciesWS [86]. The default settings were used.

### 2.4. In Silico Bacillus Toxin Screening

As performed in a previous study [77], the BLASTn online server (default settings) was used to detect toxin genes commonly associated with the *Bacillus* genus, including hemolysins (HblA, HblC, and HblD), cereulide proteins (CesA, CesB, CesC, CesD, CesH, CesP, and CesT), nonhemolytic enterotoxins (NheA, NheB, and NheC), and cytotoxin K, in the BV379 genome. In order to establish that the BLASTn screen produced both inter- and intraspecific matches, the BV379 genome was also aligned against two positive control genes: *B. subtilis* glutamyl-tRNA(Gln) amidotransferase subunit (*gatA*) and *B. cereus* methionyl-tRNA synthetase (*metG*). The sequence for the *B. cereus* cereulide gene cluster (*cesHPTABCD*) was extracted from the 270 kb plasmid pCER270 sequence (NC_010924.1) [87,88]. 

To identify proteins that are homologous to common *Bacillus* toxins, a BLASTx search was completed via the NCBI website (default settings). The protein sequences encoded by the *Bacillus* genes that were screened earlier were aligned against the query-translated BV379 genome. 

### 2.5. In Silico Biogenic Amine Screening

To assess the potential of BV379 to produce biogenic amines, the BV379 genome was screened for the nucleotide and amino acid sequences of decarboxylases, including those from common lactic acid bacteria involved in food spoilage. Nucleotide and amino acid sequence analyses were performed using BLASTn and BLASTx, respectively, under the default settings using the NCBI website [81]. 

### 2.6. In Silico Secondary Metabolite Screening 

To identity biosynthetic genes associated with secondary metabolite production, the BV379 genome was uploaded into and analyzed by the online program antiSMASH bacterial, version 6.0.1, using the default settings [89]. 

### 2.7. In Silico Virulence Factor Screening 

To determine what virulence factor (VF)-associated genes are encoded by the BV379 genome, the “full dataset” of VF-associated nucleotide sequences was downloaded from the online virulence factor database (VFDB) [90]. The full VF dataset comprises 32,170 genes for both experimentally confirmed and putative VF-associated genes from 931 medically associated bacterial strains, including *Bacillus* genes involved in adherence (e.g., BslA), antiphagocytosis, iron acquisition, regulation (e.g., AtxA), secretion systems (e.g., T7SS), and toxins (e.g., cereulide) [90]. The “core dataset” solely houses sequences of VF-associated proteins that have been experimentally verified.

Using the BLASTn algorithm [81] with local BLAST+ command line software (version 2.9.0-2) [82], the BV379 genome was aligned against the VF nucleotide dataset using the default parameters. Alignments showing <20% subject coverage were excluded from the analysis. Using the default parameters, tBLASTn was utilized to translate and align the BV379 genome to VFDB amino acid sequences, resulting in 256,833 alignments at 67,713 unique loci in the BV379 genome. Hits with <50% coverage per high-scoring segment pair (covHSP), *e* values >1, and sequence identities <60% were excluded from further analysis. Importantly, amino acid sequence alignments resulting in as low as 20% can indicate protein homology [91], so the filtered tBLASTn results represented hits with the greatest chance of homology in the BV379 genome. In cases in which regions of the BV379 genome aligned with more than one VF-associated nucleotide or amino acid sequence, the VF-associated sequence with the highest alignment bitscore was selected, and all other hits were excluded from the analysis. Hits aligning to the same locus with identical bitscores were then screened for the hit with the lowest *e* value.

### 2.8. Antimicrobial Resistance Gene and Mobile Genetic Element Screening

The BV379 genome was queried for antibiotic resistance genes using the online program Resistance Gene Identifier (RGI) [92,93]. The query was performed with the following parameters: Perfect, Strict, complete genes only, 95% identity nudge. The 95% identity nudge setting scores any loose hit with at least 95% identity as a strict hit. 

The BV379 genome was also screened for mobile genetic elements (MGEs) by aligning it to the “A CLAssification of Mobile genetic Elements” (ACLAME) database (version 0.4) using local BLASTn (default settings) [81,94]. The database consists of 125,190 bacterial plasmids and prophage and viral mobile nucleic acid sequences. Hits with less than 50% coverage were excluded from further analysis. The ISfinder server was also used to query the BV379 genome for insertion sequences [95]. The BV379 loci that aligned to MGEs or insertion sequences were manually checked for their proximity to the BV379 loci designated as antibiotic resistance genes. Sequences that were greater than 5 kb away from antibiotic resistance genes were deemed not to be associated with antibiotic resistance gene transfer [96].

### 2.9. Antibiotic Minimum Inhibitory Concentration (MIC) Assay

The minimum inhibitory concentrations (MICs) of eight common clinically relevant antibiotics, including gentamicin, vancomycin, erythromycin, clindamycin, oxytetracycline, streptomycin, kanamycin, and chloramphenicol, toward BV379 were determined by BioSciences Laboratories (Bozeman, MT, USA; Report No. 2105336-202). The MIC of each antibiotic was assessed using methods established by the Clinical and Laboratory Sciences Institute (CLSI) Document M07 [97]. Vegetative BV379 cells (3.93 × 10^6^ CFU/mL per well) were incubated with 10 different concentrations derived from a two-fold dilution series of each antibiotic in sterile nutrient broth. The concentrations for all antibiotics, except streptomycin, ranged from 0.0625 to 32 µg/mL. The concentrations for streptomycin ranged from 3.906 to 500 µg/mL. After incubation, the MIC of each antibiotic was determined based on the culture turbidity and recorded. *Staphylococcus aureus* (ATCC Accession No. 29213) and *Enterococcus faecalis* (ATCC Accession No. 29212) were tested as positive controls at 8.25 × 10^5^ and 2.96 × 10^6^ CFU/mL per well, respectively. Both positive control strains exhibited MICs within the expected control range, as outlined by the CLSI. BV379’s antibiotic susceptibility was designated based on MIC susceptibility minimums determined for *Bacillus* strains by the EFSA [98,99].

### 2.10. Blood Hemolysis Assay

To determine its ability to lyse blood cells, vegetative BV379 cells were streaked onto sheep blood agar plates. After overnight incubation at 35 °C, sheep blood agar plates were visually inspected for any discoloration. Complete depletion of color is indicative of complete hemolysis (β-hemolysis). The appearance of a darkened or green color is indicative of incomplete hemolysis (α-hemolysis). No apparent changes in the medium’s color indicated that no hemolysis occurred (γ-hemolysis).

### 2.11. Caco-2 Cell Viability Assay

To model the effects of oral BV379 supplementation on the intestinal epithelium, BV379 cell lysate was co-incubated with Caco-2 cells, an immortalized epithelial cell line of human colorectal adenocarcinoma cells, and the viability was assessed after 48 h of culture at Charles River Laboratories (Bristol, UK). The BV379 vegetative cell lysate was prepared by centrifuging an overnight culture and washing the pellet. Cells in the pellet were then processed enzymatically and mechanically via bead-beating. Intact cells were removed by filtering the sample through a 0.2 μm filter. The sterility of the cell lysate was confirmed by plating it onto TSA. A process control sample or “blank” was prepared by processing sterile media through the same centrifugation, rinsing, lysis, and filtering steps as the cell lysates. Caco-2 cells were plated onto 96-well flat-bottomed plates at 1 × 10^4^ cells/well in 100 µL volumes and allowed to adhere overnight at 37 °C and in 5% CO_2_ in a humidified chamber. BV379 lysates or “blank” process controls were added to the wells, and the Caco-2 cells were incubated for an additional 48 h. The positive control for reduced cell viability was lysed Caco-2 cells. Following the manufacturer’s guidelines, the CellTiter-Glo^®^ intracellular ATP quantification assay (Promega Corporation, Madison, WI, USA) was used to assess cell viability. A set of ATP standards were used alongside the kit for absolute quantification. A GloMax^®^ Plate reader (Promega) was used to quantify the luminescence. 

### 2.12. Caco-2 Cell Transepithelial Electrical Resistance (TEER) Assay

A transepithelial electrical resistance (TEER) assay was used to determine whether BV379 disrupts Caco-2 cell monolayer permeability (Charles River Laboratories, Portishead, UK). Caco-2 cells were seeded on Transwell^®^ inserts and allowed to generate a monolayer over 14 days. The polarized Caco-2 monolayers were then treated with a 1:5 dilution of BV379 lysate, sterile media process control, lipopolysaccharide (LPS)-positive control, or left untreated and allowed to culture for an additional 48 h. TEER was measured before treatment (0 h) and at 2, 4, 6, 24, and 48 h after treatment. TEER assays were performed in duplicate, whereby experiments were performed on separate days with independent BV379l lysate preparations. Since baseline TEER values were not consistent throughout the treatments and replicates, the TEER fold-changes were calculated relative to 0 h. 

### 2.13. Antimicrobial Activity Assay

The antimicrobial activity of BV379 was determined by a cross-streak method. BV379 was streaked from a pure liquid culture in a single line down the center of TSA plates. The plates were incubated at 35 °C for 18–24 h. Various pathogenic bacterial strains (i.e., *Bordetella bronchiseptica*, *Escherichia coli* O157:H7, *Pseudomonas aeruginosa*, *Salmonella typhimurim*, *Salmonella heidelberg*, *Salmonella enterica* subsp. *enterica* serovar *Abaetetuba*, *Listeria monocytogenes*, *Staphylococcus aureus* subsp. *aureus*, *Streptococcus agalactiae*) were then streaked in a single line perpendicular to the BV379 streaks. The plates were then incubated at 35 °C for an additional 18–24 h. Antimicrobial activity was measured as the distance (mm) between the BV379 center streak and the growth in the perpendicular streak. Each experiment was performed in triplicate.

### 2.14. Carbohydrate Utilization Screening

BV379’s carbohydrate metabolism was investigated with API^®^ 50 CH strips (bioMérieux, Marcy-l’Étoile, France) that test 49 different carbon sources. BV379 was streaked from pure liquid culture onto TSA plates and incubated at 35 °C overnight. The BV379 colonies were then suspended in an ampule of API^®^ 50 CHB/E medium (bioMérieux) until the turbidity was equivalent to 2 McFarland standard. Inoculated API^®^ 50 CHB/E medium was added to the strip cupules in 200 μL volumes, overlaid with mineral oil, and incubated for 48 h. Each cupule contained phenol red, a pH indicator that turns orange or yellow in the presence of acid. Acid production (or yellow color change) is indicative of carbon utilization. 

Further, the carbohydrase enzyme (CAZyme) gene profile of BV379 and predicted substrates were characterized by conducting DIAMOND, HMMER, and dbCAN_sub searches of the translated genome against the pre-annotated CAZyme sequence database in dbCAN3 (automated Carbohydrate-active enzyme aNnotation), an automated CAZyme annotation meta-server [100]. CAZyme annotations that were identified by fewer than two of the alignment tools were excluded from the analysis. 

### 2.15. Enzymatic Activity Screening

To determine the relative amounts of enzymes produced and secreted by BV379, a series of agar-based screens for protease, amylase, cellulase, and lipase activity was performed as previously described [21]. Briefly, BV379 cultures were diluted to a 0.5 McFarland standard and plated on skim milk (SM), starch, carboxymethylcellulose (CMC), or 1% tributyrin phenol red (TPR) agar plates and incubated at 35 °C for 18–24 h.

BV379 enzymatic activity was also investigated with API^®^ ZYM strips (bioMérieux). Each strip is composed of 20 cupules containing substrates specific for the semi-quantitative analysis of 19 different enzymatic activities. According to the manufacturer’s protocol, the colonies were suspended in saline solutions to an equivalent of 5 McFarland standard, and 65 μL was dispensed into each cupule. The API^®^ ZYM strips were covered and incubated at 35 °C for 4 h. After incubation, colorimetric indicators were added to the cupules. Following a 5 min incubation at room temperature, resulting color changes (reflecting enzymatic activities) were recorded.

### 2.16. Anaerobic Tolerance Assay

BV379 spore powder was hydrated and serially diluted in Butterfield’s buffer, plated onto TSA agar, and incubated at 35 °C overnight under three different oxygen conditions: (1) aerobic, (2) microaerobic, and (3) anaerobic. To maintain anaerobic and microaerobic conditions (2–10% oxygen), the agar plates were placed in BD GasPak™ EZ anaerobe containers (Becton, Dickinson and Company, Franklin Lakes, NJ, USA) with either BD Anaerobe sachets or CampyPouch system microaerophilic sachets, respectively. The experiments were performed in duplicate using the two batches of the BV379 spore powder (see Section 2.1). The resulting colonies were counted and used to calculate the CFU/g. The percent colony growth was calculated by comparing CFU/g to the aerobic control.

### 2.17. pH Tolerance Assay

BV379 spore powder was diluted in Butterfields’s buffer to 1 × 10^11^ CFU/mL. The spore suspension was evenly split into seven different bottles that were adjusted to a pH of 2, 3, 4, 5, 7, 9, or 10 using hydrogen chloride or sodium hydroxide. Samples (1 mL each) were withdrawn at baseline and at 30, 90, 120, 180, 240, 300, and 360 min thereafter, serially diluted in Butterfield’s buffer, and plated on TSA. The plates were incubated overnight at 35 °C. The resulting colonies were enumerated and used to calculate the CFU/mL at each timepoint. The percent survival at each time point was calculated by comparing the CFU/mL to the baseline. The data presented are the average of two trials conducted using two different batches of BV379 spore powder (Section 2.1). 

### 2.18. Bile Salt Tolerance Assay

BV379 spore powder was diluted in Butterfields’s buffer to 1 × 10^11^ CFU/mL. The spore suspension was evenly split into two different bottles. Bile salt (Sigma-Aldrich, St. Louis, MI, USA) was added to one bottle to a concentration of 0.3% (wt/vol), comparable to bile salt levels in the human small intestine [101]. The bottles were incubated at 35 °C, and samples (1 mL each) were withdrawn at baseline and at 60, 90, 120, 180, 240, 300, and 360 min thereafter, serially diluted in Butterfield’s buffer, and plated on TSA. The plates were incubated overnight at 35 °C. The resulting colonies were enumerated and used to calculate the CFU/mL at each timepoint. The percent survival at each time point was calculated by comparing the CFU/mL to the baseline. The data presented are the average of two trials conducted using two different batches of BV379 spore powder (Section 2.1). 

### 2.19. Thermostability Assay

BV379 spore powder was diluted in Butterfields’s buffer to 1 × 10^8^ CFU/mL. The spore suspension was aliquoted into 52 micro centrifuge tubes to test the spore stability at four different temperatures (i.e., 70 °C, 80 °C, 90 °C, and 100 °C) for 13 different incubation periods (i.e., 0, 5, 10, 15, 20, 25, 30, 45, 60, 90, 120, 150, and 180 min). After each respective heat treatment, each sample was cooled, and 100 µL was plated on TSA. The plates were incubated overnight at 35 °C. The resulting colonies were enumerated and used to calculate the CFU/mL at each timepoint. The percent survival at each time point was calculated by comparing the CFU/mL to baseline. The data presented are the average of two trials conducted using two different batches of BV379 spore powder (Section 2.1).

### 2.20. Statistical Analyses

For the TEER, anaerobic tolerance, thermostability, and pH stability assays, the differences among the treatments were evaluated using one-way analysis of variance (ANOVA) tests, followed by Tukey–Kramer post hoc tests. For the ATP assay, the differences were evaluated by the Kruskal–Wallis test and Dunn’s post hoc test with Bonferroni correction for multiple testing. For the bile salt tolerance assay, the differences between the treatment and control at each time point were evaluated using two-tailed *t*-tests. All tests were performed in R Studio (Version 4.0.5). The significance level was set at 0.05.

## 3. Results

### 3.1. Taxonomic Classification

**Figure 1:**
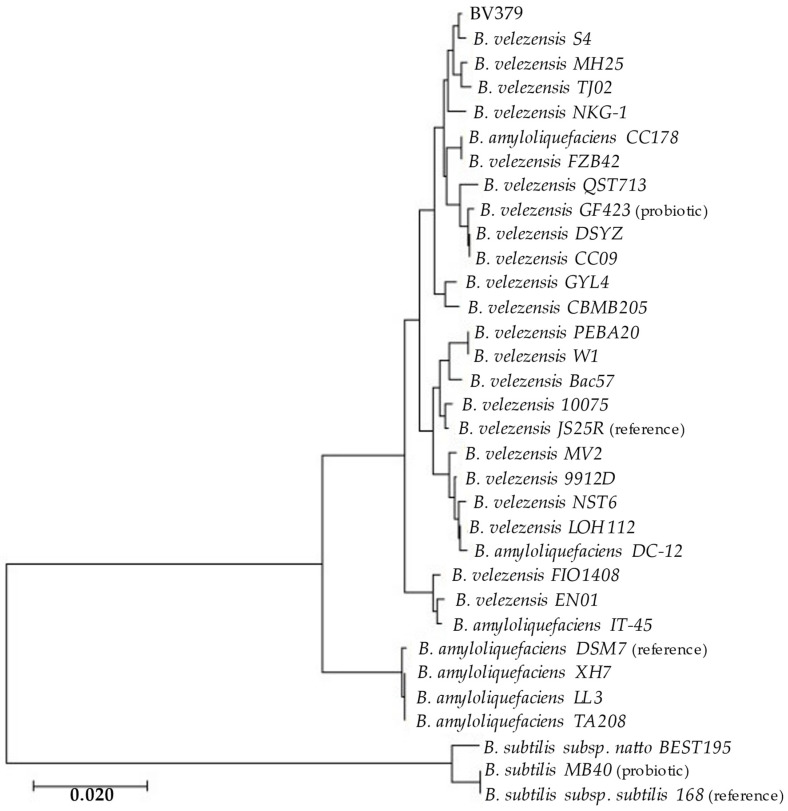
Maximum likelihood phylogenetic tree of BV379 and other *Bacillus* genomes constructed from concatenated genes: *16S rRNA*, *gyrB*, *rpoB*, *tpiA*, and *purH*, (~9300 nt). The bar indicates the nucleotide substitution rate. (reference), reference genome; (probiotic), oral probiotics [29,72,103].

To assess the taxonomic identity of BV379, multilocus sequence typing included BV379 and 32 additional *Bacillus* strains. BV379 closely aligns with *B*. *velezensis* genomes (Figure 1), including *B. velezensis* strain S4, which was isolated from biochar-treated soil [102]. 

The whole genome alignments performed using average nucleotide identity (ANI) analysis confirmed the phylogenetic findings (Table S1). BV379 had ≥97% sequence identity with all *B. velezensis* strains queried and <94% ANI with most other strains designated as *B. amyloliquefaciens*. There was <80% ANI between BV379 and the three *B*. *subtilis* strains.

### 3.2. Bacillus Toxin Screening

The nucleotide and amino acid sequences of common *Bacillus* toxin genes were aligned with the BV379 genome using BLASTn and BLASTx, respectively (Table 1 and Table S2). The BLASTn alignments between the BV379 genome and the control genes *gatA* and *metG* showed 85% identity with 100% sequence coverage and 69% identity with 95% sequence coverage, respectively (Table S2). No matches between the BV379 genome and the toxin nucleotide sequences were found. The significant sequence matches, including *NheA*, *NheB*, and *NheC* from *B*. *cereus* and *cytK* from *B*. *cereus* and *B. mycoides*, were only partial alignments that covered < 20% of the toxin sequences. The cereulide gene cluster (*cesHPTABCD*) from *B*. *cereus* aligned with 67% coverage and 67% sequence identity. 

BLASTx aligned BV379 with the positive control proteins gatA and metG (Table 1). There were no significant amino acid alignments with any of the hemolysins (HblA, HblC, and HblD), nonhemolytic enterotoxins (NheA, NheB, and NheC), or cytotoxin K (cytK). A sequence of 108 amino acids from the EntFM protein (426 amino acids long) aligned to BV379 but with only 54.6% sequence identity. All cereulide proteins, except CesD, aligned to the BV379 genome with ≤36% identity at noncontiguous genomic loci (Table S3).

### 3.3. Biogenic Amine Screening

The BLASTn analyses did not yield any significant matches between the BV379 genome and decarboxylase gene sequences (Table S4). BLASTx aligned the translated BV379 genome to five of the decarboxylase amino acid sequences with less than 30% sequence identity and less than 85% coverage over those decarboxylase sequences (Table 2). The ornithine decarboxylase protein encoded by *B. amyloliquefaciens* (MCG1032488.1) aligned to the BV379 genome with 33% identity, but only 133 amino acids out of the 711-amino-acid-long full-length protein aligned (18.7% coverage). 

### 3.4. Secondary Metabolite Screening

The BV379 genome was screened for secondary metabolites using the antiSMASH online tool, which resulted in the identification of 12 gene clusters associated with secondary metabolite synthesis (Table 3). The majority of these biosynthetic gene clusters are associated with the production of molecules with antimicrobial properties (i.e., bacillaene, macrolactin, plantazolicin, surfactin, bacilysin, difficidin, bacillibactin, fengycin, and butirosin). However, only 7% of the butirosin A/B operon (two of 26 genes) was detected in the BV379 genome.

### 3.5. Virulence Factor Screening

Similar to the antiSMASH results, the alignments between the BV379 genome and the VFDB nucleotide and amino acid datasets suggested the presence of genes/proteins involved in bacillibactin synthesis (Tables S5 and S6). Notably, four proteins involved in polyglutamate synthesis, three proteins involved in bacillibactin synthesis, and one hemolysin were identified in the translated BV379 genome (Table S6). Several other proteins involved in capsule formation, stress survival, adherence, nutritional/metabolic pathways, and translational/transcriptional regulation were also identified.

### 3.6. Antimicrobial Resistance Gene and Mobile Genetic Element Assay

To address the potential of BV379 to horizontally transfer antimicrobial resistance to other microbes, including pathogens, the BV379 genome was screened for antimicrobial resistance genes and flanking mobile genetic elements. Five antimicrobial resistance genes were detected in the BV379 genome, including four small multidrug-resistance (SMR) antibiotic efflux pump genes that confer resistance against disinfecting agents and antiseptics and the *clbA* gene, which provides resistance against lincosamides (Table 4). 

Within the BV379 genome, 209 unique loci aligned with known mobile MGEs (Table S7). On the basis of the ISfinder results, 63 loci in the BV379 genome aligned with insertion sequences (ISs), including ISBsu1 (NC_014479), ISLse4 (CP063071.1), ISBt2 (CP001186), ISBce5 (NC_006274), ISTli1 (NC_022084), ISLmo1 (NC_003210), and ISErh1 (direct submission to ISfinder) (Table S8). None of the loci aligning to MGEs or ISs were within 5 kb of any of the five antimicrobial genes (Table S9). 

### 3.7. Antibiotic Minimum Inhibitory Concentration (MIC) Assay

BV379 was confirmed to be sensitive to eight medically relevant antibiotics based on minimum values as determined by the European Food Safety Authority (EFSA) (Table 5).

### 3.8. Blood Hemolysis Assay

After incubation of BV379 on sheep blood agar plates for 24 h, no change in the color of the agar or hemolysis was observed on or near the BV379 colonies (Figure S1). 

### 3.9. Caco-2 Cell Viability and Transepithelial Electrical Resistance (TEER) Assays

**Figure 2:**
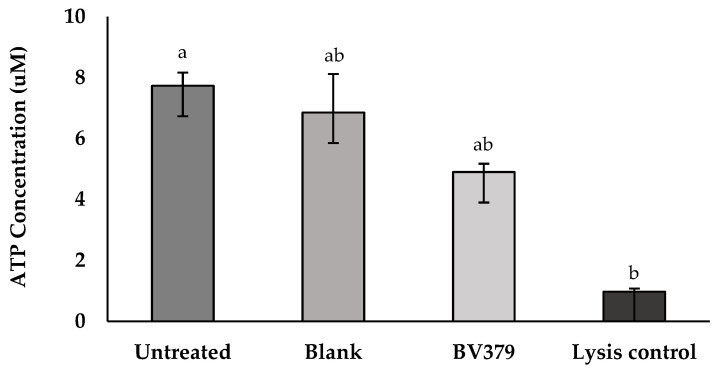
Effect of 48-h BV379 lysate on Caco-2 cell viability based on intracellular ATP concentrations. There were two controls: untreated Caco-2 cells (negative control) and a 100% lysis-positive control. The blank process treatment was uninoculated media processed identically to BV379 lysates. Data are expressed as the means ± standard deviation of technical triplicates. Significant differences (*p* < 0.05) between samples are denoted by unshared lower-case letters (a, b).

To determine whether BV379 affects intestinal epithelial cell viability or monolayer barrier integrity in vitro, ATP and TEER assays were carried out with Caco-2 cells. In the ATP assay, intracellular ATP concentrations are directly proportional to the cell viability. After exposure to BV379 lysate for 48 h, intracellular ATP concentrations were determined using a standard curve (Figure 2). The BV379 lysate treatment did not significantly affect ATP concentrations as compared to the process blank and untreated controls (*p* > 0.14). The ATP concentrations of the BV379 lysate-treated and process blank-treated cells were not significantly different from the lysis control (*p* > 0.14). 

**Figure 3:**
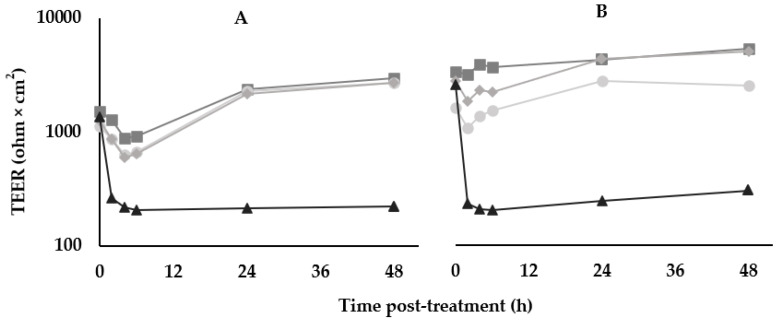
Effects of BV379 lysates on Caco-2 cell monolayer transepithelial electrical resistance (TEER) in duplicate experiments (*n* = 2, panels (**A**,**B**)). TEER was measured at before treatment (0 h) and at 2, 4, 6, 24, and 48 h after treatment. ■, Untreated Caco-2 cells; ◆, “blank”/uninoculated lysate processing control; ●, BV379 lysate treatment; ▲, LPS treatment (positive control). Values on the y-axis are plotted on a logarithmic scale.

For the TEER disruption assays, Caco-2 monolayers were treated with BV379 lysate, a media blank, an LPS-positive control treatment, or were left untreated (Figure 3). At 48 h treatment, no significant differences in the fold-change values between the untreated control, blank process control, and cells treated with BV379 lysate were observed (*p* > 0.9). The LPS-positive control reduced TEER values compared to the other treatments (*p* < 0.05). 

### 3.10. Antimicrobial Activity Assay

Cross-streak agar assays were used to determine the antimicrobial activity of BV379. BV379 inhibited the growth of all microorganisms tested (Table 6). 

### 3.11. Carbohydrate Utilization Screening

The API^®^ CH 50 strip testing showed that BV379 was able to utilize 23 of the 49 different carbohydrates tested (Table 7). The following carbon sources showed no evidence of metabolism by BV379: erythritol, D-arabinose, L-xylose, D-adonitol, methyl-β-D-xylopyranoside, D-galactose, D-mannose, L-sorbose, L-rhamnose, dulcitol, methyl-α-D-mannopyranoside, D-melibiose, inulin, D-melezitose, xylitol, gentiobiose, D-turanose, D-lyxose, D-tagatose, D-fucose, L-fucose, D-arabitol, L-arabitol, potassium gluconate, potassium 2-ketogluconate, and potassium 5-ketogluconate. Likewise, 105 genes were identified as enzymes involved in carbohydrate metabolism (CAZymes) using dbCAN3 annotation (Table S10). There were 20 different predicted potential CAZyme substrates across 48 of these genes, including arabinan, β-fucosides, β-galactan, β-glucan, β-mannan, cellulose, cephalosporin C, chitin, chitooligosaccharide, chitosan, fructan, host glycan, pectin, peptidoglycan, polyphenol, raffinose, starch, sucrose, trehalose, and xylan (Table S11).

### 3.12. Enzymatic Activity Screening

Both agar-based screens and API^®^ ZYM strips were used to determine the enzymatic activity of BV379. General amylase, cellulase, and protease activities were observed in the agar-based screens, but lipolytic activity was not observed (Table S12). In contrast, the incubation with the API^®^ ZYM test strips indicated that BV379 exhibits lipolytic activity (esterase and esterase lipase) toward glycerol esters with short acyl chains (Table 8). Lipolysis of compounds with longer acyl chains was not detected. 

### 3.13. Anaerobic Tolerance Assay

**Figure 4:**
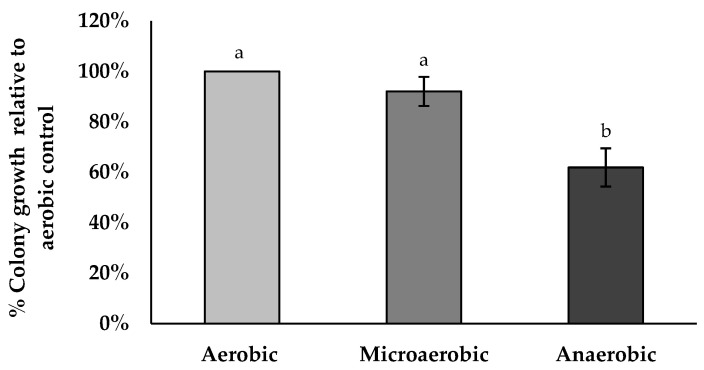
BV379 colony growth on tryptic soy agar plates incubated at 35 °C under aerobic, microaerobic, or anaerobic conditions. Experiments were performed with two biological replicates. Data are expressed as the means ± standard deviation of biological duplicates. Significant differences (*p* < 0.05) between samples are denoted by unshared lower-case letters (a, b).

BV379 was plated onto TSA agar plates and incubated overnight at 35 °C under aerobic, microaerobic, or anaerobic conditions. The percent colony viability was significantly lower in anaerobic growth conditions relative to aerobic conditions and microaerobic conditions (*p* < 0.05). The percent colony viability in microaerobic conditions was not significantly different than aerobic conditions (*p* = 0.43) (Figure 4).

### 3.14. pH Tolerance Assay

**Figure 5:**
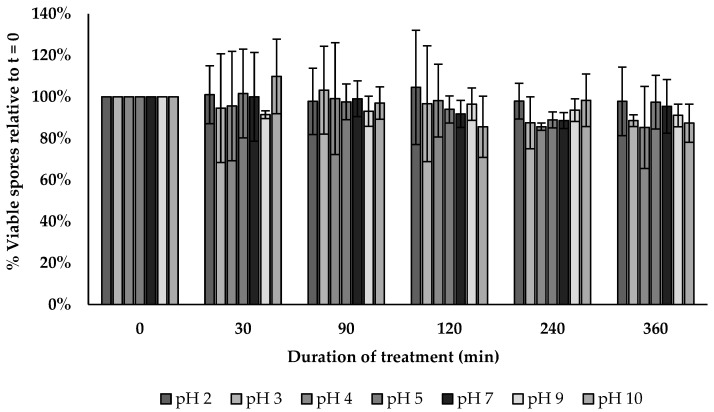
BV379 spore viability across a broad range of pH. Data are expressed as the means ± standard deviation of biological duplicates.

The BV379 spore viability across a pH range of 2–10 for up to 6 h was similar across treatments per exposure time (*p* > 0.5) (Figure 5). 

### 3.15. Bile Salt Tolerance Assay

**Figure 6:**
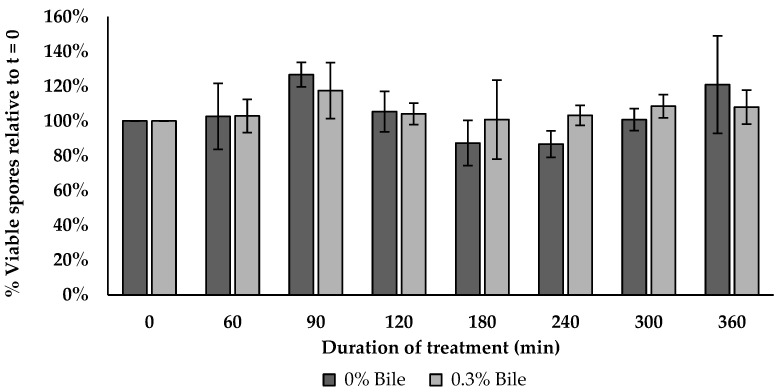
BV379 spore viability in 0.3% bile salt over 6 h incubation. Data are expressed as the means ± standard deviation of biological duplicates.

The BV379 spore viability was similar between the control and bile salt treatment at all timepoints tested (*p* > 0.134) as shown in Figure 6.

### 3.16. Thermostability Assay

**Figure 7:**
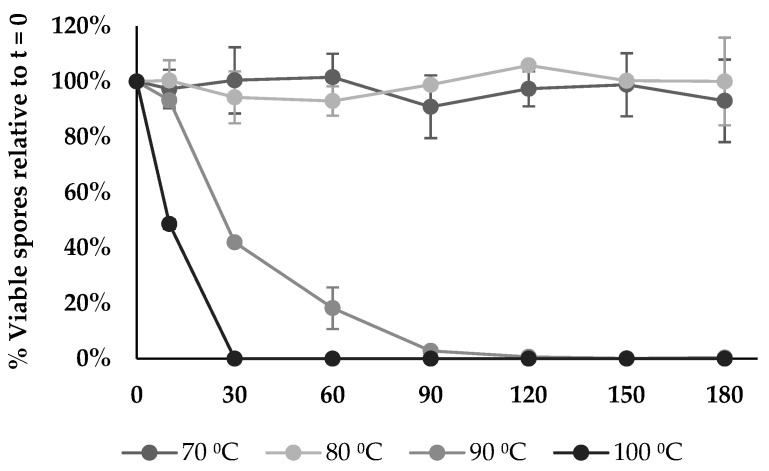
BV379 spore viability at different temperatures over time. Data are expressed as the means ± standard deviation of biological duplicates.

The spore thermostability of BV379 was tested at 70, 80, 90, and 100 °C for 3 h. At 70 °C and 80 °C, spore viability did not differ from the baseline throughout the time course (*p* > 0.21) (Figure 7). The spore viability at 90 °C remained stable until 30 min, and no viability was observed by 150 min. At 10 min, spore viability at 100 °C was significantly reduced to 49% relative to baseline (*p* < 0.05), and by 30 min, no spores were viable (*p* < 0.05). 

## 4. Discussion

This study describes a series of preclinical in silico and in vitro screens that assess the safety and metabolic capabilities of the oral probiotic candidate BV379. Further, this work characterized the ability of BV379 to tolerate various stressors associated with food manufacturing (i.e., high temperatures), as well as the microenvironments of the human GI tract (i.e., low pH and presence of bile salts). First, BV379 was confirmed to belong to the taxonomic species *B. velezensis*, as it showed >96% sequence identity with 22 other *B. velezensis* strains (Table S1). An average nucleotide identity of 95% is the standard cutoff for shared species identity [104,105].

In silico safety screening of the BV379 genome began with screening for common *Bacillus* toxins. It was found that all proteins belonging to the cereulide-encoding operon (cesPTABCD), except for CesD, aligned to the translated BV379 genome with no more than 40% sequence identity. It is not surprising that BV379 would encode proteins homologous to cereulide proteins, as they belong to common and essential protein families, including hydrolases (CesH), thioesterases (CesT), ABC transporters (CesC), nonribosomal peptide synthases (CesA and CesB), and phosphopanthetheinyl transferases (CesP) [106]. The cesPTABCD operon is transcribed into a single 23 kb polycistronic mRNA [106]. Given that the cereulide alignments were noncontiguous, it is likely that BV379 does not encode any functional cereulide genes. 

Bacterially produced biogenic amines, such as histamine and tyramine, can have negative clinical effects after oral consumption [107,108]. Decarboxylases are the primary bacterial enzymes responsible for converting amino acids to biogenic amines and can be the cause of food spoilages [108]. The BV379 genome was screened for nucleotide and amino acid sequences of decarboxylases from available *Bacillus* strains and common lactic acid bacteria involved in food spoilage. The translated BV379 genome aligned to five decarboxylase amino acid sequences with <35% sequence identity and <85% coverage. On the basis of these results, it is not likely that BV379 is capable of synthesizing a functional decarboxylase involved in biogenic amine production. Higher polyamines, such as spermine and spermidine, are synthesized from putrescine by decarboxylases [109,110]. Because BV379 does not encode for any complete ornithine or agmatine decarboxylases involved in putrescine production, it is also likely that BV379 cannot produce spermine and spermidine.

As an additional measure to identify deleterious genes, BV379 was screened for secondary metabolites and virulence factors. The analysis of the BV379 genome resulted in the full alignment of 11 gene clusters associated with secondary metabolite synthesis (Table 3). The majority of these biosynthetic gene clusters are associated with the production of molecules with antimicrobial properties. Antimicrobial metabolites such as bacteriocin peptides (e.g., surfactin, fengycin, iturin, and bacillomycin) and polyketides (e.g., macrolactin, difficidin, and bacilysin) have been reported in *B. velezensis* isolates [61,111,112], and up to 10% of their genomes are devoted to genes involved in antimicrobial production [42,58]. Importantly, two *B*. *velezensis* strains that were independently isolated from healthy human stool (LOH112 and ADS024) encode for the same antiSMASH-identified biosynthetic gene clusters as BV379 (e.g., bacillaene, macrolactin H, difficidin, surfactin, bacillibactin, fengycin, and bacilysin), with the exception of plantazolicin [57,58]. 

The virulence factor screen highlighted three proteins involved in bacillibactin synthesis, one hemolysin, and three proteins involved in polyglutamate synthesis. While polyglutamate enhances the ability of *Bacillus anthracis* and *Staphylococcus epidermidis* to evade the innate immune responses of their hosts [113,114], *B. sonorensis*-derived poly-γ-glutamic acid can reduce *Staphylococcus aureus* and *Escherichia coli* growth [115]. Many commensal *Bacillus* strains, including natto strains and other strains isolated from fermented foods (e.g., cheongkukjang, doenjang, and kochujang) also produce polyglutamate [116,117]. *Bacillus velezensis* strains, specifically, have an enormous bioactive secondary metabolite repertoire that includes molecules that are effective against pathogenic bacteria and fungi. In summary, the secondary metabolite biosynthetic gene clusters and virulence factors identified in BV379 are widely present throughout *Bacillus* strains, including strains isolated from human stool, and likely do not represent a safety concern.

One potential concern, however, was the presence of a hemolysin-encoding gene in the BV379 genome. Hemolysin genes are common to *Bacillaceae* probiotic strains [118], including *Bacillus subtilis* BS50, which was shown to be safe in healthy adults following six weeks of daily oral supplementation [21,77]. To address the concern of potential hemolytic activity, BV379 was plated and incubated on a blood agar medium, and it was determined that BV379 does not exhibit discernable hemolytic activity. Additionally, there is low risk of an oral probiotic translocating into the bloodstream, a phenomenon only sparsely observed in preterm infants and intensive care unit patients [119,120]. Moreover, it is encouraging that BV379 showed no deleterious effects on human intestinal epithelial cell viability or monolayer permeability in cell culture (Figure 3). An important limitation regarding these cell culture assays, however, is that human cells were incubated with lysates of BV379 cells that were pelleted from liquid culture and separated from the supernatant. *Bacillus* species are highly efficient at secreting enzymes, antimicrobials, and other bioactive molecules [121,122]. Although it is possible that cell lysates contain a subset of these secreted molecules at some concentration, future studies of supernatant interactions with human cells are likely to be insightful.

To address the potential of BV379 to horizontally transfer antimicrobial resistance to other microbes, the BV379 genome was screened for antimicrobial resistance genes and flanking mobile genetic elements. Four genes that confer resistance against disinfecting agents and antiseptics and one gene that provides resistance against lincosamides were detected in the BV379 genome (Table 6). None of the genomic loci aligning to MGEs or ISs were within 5 kb of any the loci of the five antimicrobial genes, indicating they are not capable of horizontal transfer [96]. BV379 was susceptible to all eight medically relevant antibiotics based on MIC thresholds established by the EFSA [123]. BV379 does not exhibit any concerning antibiotic resistance characteristics and should be considered low risk for introducing broad spectrum antibiotic resistance factors into the gastric microbiome when administered orally.

Using a combination of in silico screens, agar-based assays, and biochemical API^®^ strip assays, BV379 was shown to utilize a wide range of carbohydrates, including cellobiose, raffinose, xylans, and starch, and to exhibit robust protease activity yet limited lipase activity. On the basis of these data, BV379 shows promise as an oral probiotic to support digestion of dietary protein and specific dietary fibers and/or nonstarch polysaccharides.

Also encouraging is the ability of BV379 spores to survive conditions typical of the human GI tract. For example, BV379 showed tolerance to microaerobic conditions, a wide range of pH, and bile salt. A primary stressor that an orally consumed probiotic will encounter in the GI tract is gastric acidity where pH can be as low as pH 1–2 [124]. After traversing from the stomach into the duodenum of the small intestine, the next stressor is bile salt [101]. Bile salts are particularly toxic to bacteria because they can act as detergents and disrupt bacterial cell walls [125]. Interestingly, bile salts can also promote germination of bacterial spores [126]. Likewise, probiotic spores have previously been shown to germinate within the small intestine [127]. As partially germinated probiotic cells descend to the lower GI tract, another possible microbial deterrent is hypoxia. Within the large intestine, there is an oxygen gradient whereby oxygen is present at microaerobic concentrations (7–8% oxygen) at the lamina propria and proceeds to <1% within the lumen [128,129]. While BV379 colony growth was reduced to 62% in anaerobic conditions relative to aerobic conditions, it was not significantly reduced in microaerobic conditions (2–10% oxygen), supporting the ability of BV379 to survive throughout the oxygen gradient of the intestine. 

Furthermore, a probiotic’s ability to withstand high temperatures can be instrumental for formulation into foods or supplements that undergo thermal processing. For example, inclusion temperatures in the gummy manufacturing process can range from 65–77 °C for gelatin- and other protein-based gummies, while starch- or pectin-based gummies are processed at higher temperatures (e.g., >100 °C) [130]. Processing temperatures above 45–50 °C are considered detrimental to the viability of non-spore-forming probiotics [131]. Given that BV379 spores could easily withstand extended exposure to temperatures as high as 80 °C, BV379 would be well-suited as an addition to gelatin-based gummy supplements [124]. Further, depending on the specific pasteurization process employed (e.g., non-higher-heat shorter time (HHST) processes), BV379 could be compatible with pasteurized foods and beverages [132].

Additional preclinical studies are warranted to investigate candidate mechanisms of BV379 action in the human gut, including bifidogenic activity, antioxidant activity, human toll-like receptor modulation, mucin adhesion, human epithelial cell association, and luminal biofilm formation. Another fundamental next step is to investigate safety and efficacy in humans, and the first in-human clinical trial of oral BV379 supplementation recently completed participant evaluation [133]. 

## 5. Conclusions

Spore-forming bacterial strains from the species *Bacillus subtilis* and *Heyndrickxia coagulans* are commonly formulated into dietary supplements and foods. We identified a new strain, BV379, from the related species *Bacillus velezensis* in a systematic screen for novel probiotics. Altogether, a series of preclinical in silico and in vitro screens assessing the safety, metabolism, and survivability of the probiotic candidate *Bacillus velezensis* strain BV379 supports it safe use and functional potential in human probiotic applications.

## Figures and Tables

**Table 1:** BLASTx alignments of known *Bacillus* toxin proteins and the translated BV379 genome.

Protein	Organism	Accession	Max Score	*e* Value	% Identity
GatA	*B. subtilis*	NP_388550.1	6600	0	96.5
MetG	*B. cereus*	WP_079994147.1	1019	0	74.3
EntFM	*B. cereus*	AAX14641.1	112	3 × 10^−26^	54.6
CesA	*B. cereus*	WP_002081542.1	968	0	35.5
CesB	*B. cereus*	WP_000953496.1	653	0	34.8
CesC	*B. cereus*	WP_000590108.1	139	3 × 10^−36^	32.6
CesH	*B. cereus*	WP_000291846.1	32.7	8 × 10^−1^	20.9
CesP	*B. cereus*	WP_000680399.1	128	6 × 10^−33^	34.4
CesT	*B. cereus*	WP_000764755.1	60.8	1 × 10^−17^	30.7
CesD	*B. cereus*	WP_001008264.1	No significant similarity found		
cytK	*B. mycoides*	AAW56196.1			
cytK	*B. cereus*	AAY84864.1			
NheA	*B. mycoides*	AAZ82480.1			
NheB	*B. mycoides*	AAZ82481.1			
NheC	*B. mycoides*	AAZ82482.1			
NheA	*B. cereus*	ABI52601.1			
NheB	*B. cereus*	ABI52602.1			
NheC	*B. cereus*	ABI52603.1			
NheA	*B. cereus*	CBL95107.1			
NheA, partial	*B. thuringiensis*	ACM18211.1			
NheB	*B. thuringiensis*	ACM18212.1			
NheC, partial	*B. thuringiensis*	ACM18213.1			
HblD	*B. cereus*	AFN08801.1			
HblC	*B. cereus*	AFN08807.1			
HblA	*B. cereus*	AII31101.1			
HblD	*B. licheniformis*	AIR07774.1			
HblA	*B. licheniformis*	AIR07775.1			
cytK	*B. licheniformis*	AIS75096.1			

**Table 2:** BV379 BLASTx alignments with biogenic amine-associated proteins.

Protein	Products	Species	Accession	Max Score	*e* Value	% Identity
Ornithine decarboxylase	Putrescine	*P. wasatchensis*	UHY45096.1	83.6	4 × 10^−17^	23.4%
Ornithine decarboxylase	Putrescine/ Cadaverine	*B. amyloliquefaciens*	MCG1032488.1	74.3	3 × 10^−14^	33.8%
Ornithine/ Lysine decarboxylase	Putrescine	*P. wasatchensis*	WP_044011228.1	69.7	6 × 10^−13^	27.2%
Lysine decarboxylase 1	Putrescine	*E. coli* str. *K-12*	NP_418555	82.8	6 × 10^−17^	25.3%
Lysine decarboxylase 2	Cadaverine	*E. coli* str. *K-12*	NP_414728	79.7	5 × 10^−16^	25.4%
Lysine decarboxylase	Cadaverine	*B. amyloliquefaciens*	MCG1033704.1	75.1	1 × 10^−14^	25.8%
Histidine decarboxylase	Histamine	*L. parabuchneri*	CUA78690.1	No significant similarity found		
Histidine decarboxylase	Histamine	*B. licheniformis*	BAJ05382.1			
Tyrosine decarboxylase	Tyramine	*L. brevis*	WP_021742084.1			
Tyrosine decarboxylase	Tyramine	*B. cereus*	WP_098989318.1			
Agmatine deiminase	Putrescine	*L. brevis*	ABS19477.1			
Agmatine deiminase	Putrescine	*B. massiliogorillae*	WP_042353033.1			

**Table 3:** BV379 secondary metabolites predicted by antiSMASH.

Cluster Type	Most Similar Cluster	% Identity
NRPS, Betalactone, Transat-PKS	Fengycin	100%
Transat-PKS, NRPS-Like, NRPS, T3PKS	Bacillaene	100%
Transat-PKS	Macrolactin H	100%
Other	Bacilysin	100%
Ripp-Like, NRPS	Bacillibactin	100%
Transat-PKS	Difficidin	93%
LAP, RRE-Containing	Plantazolicin	91%
NRPS-Like, NRPS	Surfactin	91%
PKS-Like	Butirosin A/Butirosin B	7%
Terpene	-	-
Terpene	-	-
T3pks	-	-

antiSMASH, antibiotics and Secondary Metabolite Analysis Shell web server [89]; NRPS, nonribosomal peptide; PKS, polyketide synthase.

**Table 4:** Antibiotic resistance gene detection via RGI.

ARO Term (Gene)	AMR Gene Family	Drug Class	% Identity	% Length	RGI Criteria
*qacG*	SMR antibiotic efflux pump	Disinfecting agents and antiseptics	42.5	113.1	Strict
*qacJ*	SMR antibiotic efflux pump	Disinfecting agents and antiseptics	44.9	112.2	Strict
*qacJ*	SMR antibiotic efflux pump	Disinfecting agents and antiseptics	37.4	97.2	Strict
*qacJ*	SMR antibiotic efflux pump	Disinfecting agents and antiseptics	56.3	76.6	Strict
*clbA*	Cfr 23S ribosomal RNAmethyltransferase	Lincosamide; Streptogramin; Streptogramin A; Oxazolidinone; Phenicol; Pleuromutilin	99.7	100	Strict

ARO, Antibiotic Resistance Ontology; RGI, Resistance Gene Identifier; SMR, small multidrug resistance.

**Table 5:** In vitro minimum inhibitory concentrations (MICs) of antibiotics for BV379.

Antibiotics	Type	MIC (µg/mL)	EFSA MIC (µg/mL) Resistance Threshold ^a^
Chloramphenicol	Phenicol	1.00	8
Clindamycin	Macrolides, lincosamides	0.50	4
Erythromycin	Macrolides, lincosamides	<0.0625	4
Gentamicin	Aminoglycosides	<0.0625	4
Kanamycin	Aminoglycoside	0.500	8
Streptomycin	Aminoglycoside	3.906	8
Oxytetracycline	Tetracycline	0.125	8
Vancomycin	Glycopeptide	0.25	4

^a^ EFSA MIC thresholds for resistance are specific to *Bacillus* strains [93,94].

**Table 6:** Antimicrobial activity of BV379 ^a^.

Gram Reaction	Strains	Activity
Gram-positive	*Bordetella bronchiseptica*	++
	*Escherichia coli O157:H7*	+
	*Pseudomonas aeruginosa*	+
	*Salmonella typhimurim*	++
	*Salmonella heidelberg*	+
	*Salmonella* enterica subsp. *enterica* serovar *Abaetetuba*	+
Gram-negative	*Listeria monocytogenes*	++
	*Staphylococcus aureus* subsp. *aureus*	+
	*Streptococcus agalactiae*	+

^a^ The number of plus signs indicates the relative size of the clearing zones (i.e., antimicrobial activity). +, Clearing zone < 5 mm; ++; clearing zone > 5 mm and < 10 mm. Each experiment was repeated at least three times, and the mean values were used for scoring.

**Table 7:** Carbohydrates utilized by BV379 in cell culture ^a^.

Carbohydrate	Putative Genetic Mediator(s)
Amygdalin	N/A
Arbutin	*arbF*
D-Cellobiose	*arbF*, *bglP*, *chbB*
D-Fructose	*SORD*, *xylA*, *ptf1*, *scrK*
D-Glucose	*pgm*, *pgi*
D-Lactose (bovine origin)	*araQ*, *araN*, *araP*
D-Maltose	*glva*, *MGAM*
D-Mannitol	N/A
D-Raffinose	*GLA*, *sacA*, *msmE*, *msmF*, *msmG*
D-Ribose	*rbsA*, *rbsB*, *rbsC*
D-Saccharose (sucrose)	*sacA, malL, MGAM*
D-Sorbitol	*SORD*
D-Trehalose	*treC*
D-Xylose	*xylA*, *lyxA*, *xylB*
Esculin + ferric citrate	N/A
Glycerol	*glpk*, *GCY1*
Glycogen	*amyA*
Inositol	*iolG*
L-Arabinose	*AraA*, *AraB*
Methyl-α-D-glucopyranoside	N/A
N-Acetylglucosamine	*murA*
Salicin	*arbF*
Starch (amidon)	*amyA*

^a^ Carbon utilization as determined by API^®^ CH 50 strip testing. N/A, indicates that no genes associated with metabolism of the corresponding carbohydrate were found in the RASTtk/dbCAN3-annotated BV379 genome.

**Table 8:** API^®^ ZYM test results for BV379 ^a^.

Enzyme	Result	Gene
Alkaline phosphatase	+	Present
Esterase (C 4)	+	Present
Esterase lipase (C 8)	+	Present
Lipase (C 14)	−	N/A
Leucine arylamidase	+	Present
Valine arylamidase	−	Present
Cystine arylamidase	−	N/A
Trypsin	−	N/A
D-chymotrypsin	−	N/A
Acid phosphatase	+	Present
Naphthol-AS-BI-phosphohydrolase	+	N/A
α-Galactosidase	−	Present
β-Galactosidase (lactase)	−	Present
β-Glucuronidase	−	N/A
α-Glucosidase	+	Present
β-Glucosidase	−	Present
N-acetyl-β-glucosaminidase	−	N/A
α-Mannosidase	−	N/A
α-Fucosidase	−	N/A

^a^ The RASTtk-annotated BV379 genome was screened for the presence of the corresponding gene. +, enzymatic activity observed; –, enzymatic activity not observed; N/A, indicates that no corresponding enzyme-encoding genes were found in the RASTtk-annotated BV379 genome.

## Data Availability

Data are contained within the article.

## Supplementary Materials

The following supporting information can be downloaded at: https://www.mdpi.com/article/10.3390/microorganisms12030436/s1, Figure S1: BV379 hemolysis assay; Table S1: Average nucleotide identity (ANI) between *Bacillus velezensis* BV379 and 32 other *Bacillus* strains; Table S2: Summary of BLASTn screening results for known *Bacillus* toxin genes in BV379; Table S3: BLASTx alignments between the BV379 genome and cereulide proteins. The columns “Alignment Start” and “Alignment End” correspond to the BV379 genomic loci of each alignment; Table S4: Summary of BV379 BLASTn screening results for genes involved in biogenic amine production; Table S5: Virulence-factor-associated genes predicted in the BV379 genome via BLASTn; Table S6: Virulence-factor-associated amino acid sequences that aligned to the translated BV379 genome via tBLASTn; Table S7: BV379 genome BLASTn alignment results to the ACLAME (a CLAssification of Mobile genetic Elements) database; Table S8: BV379 genome alignment results to the ISfinder database; Table S9: BV379 genome alignment results to the Comprehensive Antibiotic Resistance Database (CARD); Table S10: CAZyme gene profile of BV379; Table S11: CAZyme gene and substrate profile of BV379 as predicted by dbCAN_sub; Table S12: Agar-based enzymatic activity of BV379.
