# Supplementary material for: Genetic and Phenotypic Characterization of Bacillus velezensis Strain BV379 for Human Probiotic Applications
Source: Microorganisms. 2024 Feb 21;12(3):436. doi: 10.3390/microorganisms12030436 (PMC10974050; doi:10.3390/microorganisms12030436)
Supplement: Supplementary file 1 [file microorganisms-12-00436-s001.zip › Supplemental_material/Supplemental Figure S1.pdf]

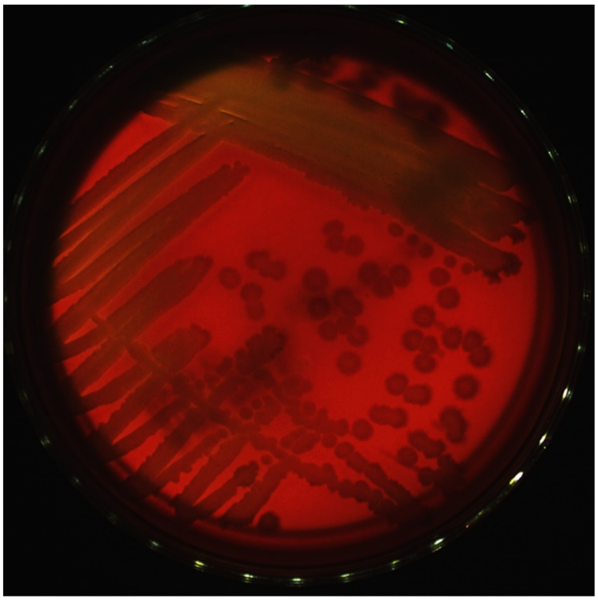

Figure S1. BV379 hemolysis assay. BV379 was streaked onto sheep blood agar plates. After overnight incubation at 35 °C, the agar was inspected for α- or β-hemolysis. An indiscernible change in the color of the agar indicates that no hemolysis occurred (i.e., γ-hemolysis). BV379 exhibits γ-hemolysis.
